# The societal cost of heroin use disorder in the United States

**DOI:** 10.1371/journal.pone.0177323

**Published:** 2017-05-30

**Authors:** Ruixuan Jiang, Inyoung Lee, Todd A. Lee, A. Simon Pickard

**Affiliations:** 1 Center for Pharmacoepidemiology and Pharmacoeconomics Research and Department of Pharmacy Systems, Outcomes and Policy, University of Illinois at Chicago, College of Pharmacy, Chicago, IL, United States of America; 2 Department of Medical Research, China Medical University Hospital, China Medical University, Taichung, Taiwan; Old Dominion University, UNITED STATES

## Abstract

**Objective:**

Heroin use in the United States has reached epidemic proportions. The objective of this paper is to estimate the annual societal cost of heroin use disorder in the United States in 2015 US dollars.

**Methods:**

An analytic model was created that included incarceration and crime; treatment for heroin use disorder; chronic infectious diseases (HIV, Hepatitis B, Hepatitis C, and Tuberculosis) and their treatments; treatment of neonatal abstinence syndrome; lost productivity; and death by heroin overdose.

**Results:**

Using literature-based estimates to populate the model, the cost of heroin use disorder was estimated to be $51.2 billion in 2015 US dollars ($50,799 per heroin user). One-way sensitivity analyses showed that overall cost estimates were sensitive to the number of heroin users, cost of HCV treatment, and cost of incarcerating heroin users.

**Conclusion:**

The annual cost of heroin use disorder to society in the United States emphasizes the need for sustained investment in healthcare and non-healthcare related strategies that reduce the likelihood of abuse and provide care and support for users to overcome the disorder.

## Introduction

The widespread availability and use of heroin has created a major public health crisis in the United States. The number of heroin users doubled from 2000 to 2013, rising from 1.0 per 1,000 persons in 2000 to 2.0 per 1,000 persons in 2013; heroin overdose deaths have also more than tripled since 2002.[1–4] There are tremendous personal and social costs to heroin use disorder, and an economic argument can be made for the need to invest in education, prevention and rehabilitation services, as well as legislative reform to address this issue. Heroin users are less productive than other society members due to premature death; enrollment in drug treatment centers; and drug-related hospitalizations, absenteeism, and unemployment.[5–7] High rates of criminal activity and incarceration among heroin users further exacerbate the societal economic burden due to direct costs (e.g. value of stolen property and cost of incarceration) as well as productivity loss during incarceration.[8, 9] Additionally, heroin use, specifically via injection, is associated with several chronic infectious diseases—Hepatitis C (HCV), Hepatitis B (HBV), HIV-AIDS, and Tuberculosis (TB).[10–12] The treatment costs for these chronic conditions are substantial; for instance, HIV treatment is estimated to cost more than $300,000 over a lifetime.[13]

The economic impact of heroin use disorder (the contemporarily accepted term for heroin addiction) to society in the context of the ongoing heroin epidemic is unclear. Characterization of the current economic burden of heroin use disorder is important to understanding the magnitude of its impact, which can subsequently inform the extent to which resources should be directed towards mitigating the devastating impact of heroin use disorder. Thus, the aim of this paper is to estimate the annual societal cost of heroin use disorder in the United States in 2015 US dollars; specifically, we sought to calculate the amount of money society would save if heroin use disorder was eliminated.

## Methods

### Model design

**Fig 1:**
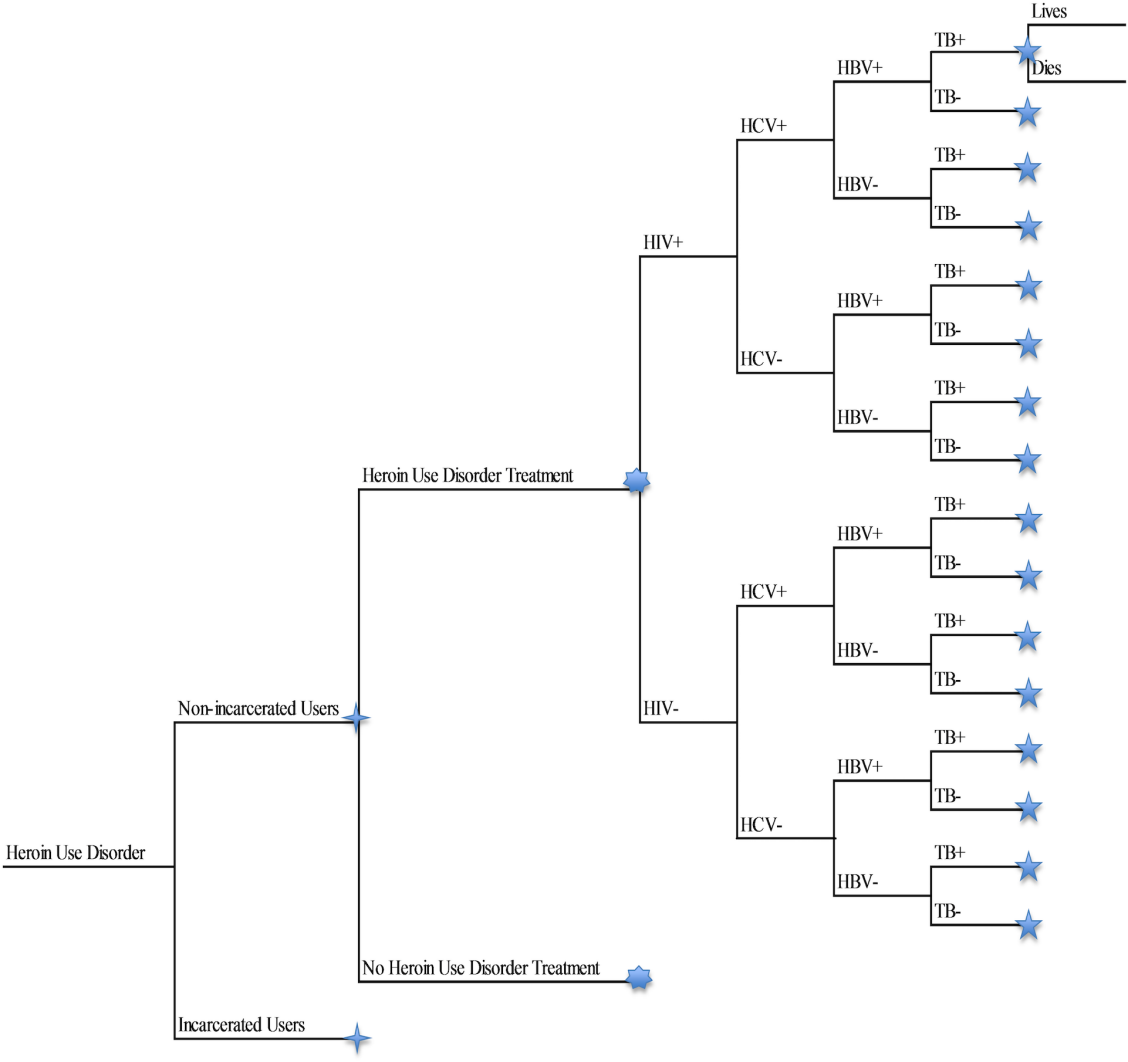
Simplified structure of the cost-analytic model. The cost-analytic model was used to assist in the calculation of a prevalence based estimate of the annual cost of heroin use disorder in the United States. Thus, only prevalent cases were included in the model. Not shown in model: Proportion of treatment applied for each chronic infectious health state.

An analytic model was designed in TreeAge Pro 2017 (Williamstown, MA) to calculate the societal cost of prevalent heroin use disorder using the most recently available data (Fig 1). The model was structured using mutually exclusive ‘health states’ that related to all possible combinations of the following: incarceration or non-incarceration; chronic infectious diseases (HIV, HBV, HCV and TB, including no chronic infectious diseases); and health state (heroin use disorder and infectious diseases) treatment or no treatment. [14–17]

In the model, the overall population with heroin use disorder was stratified into incarcerated population and non-incarcerated populations. Throughout this study, the phrase “heroin user(s)” refers to users with heroin use disorder as we have operationalized it unless as a citation to another study, for which we defer to the original researchers' definitions; the same is also true with the application of “heroin use” in this study. Subsequently, the model was structurally identical and only differentiated by the prevalence rates of health state occurrence. Each incarceration group was then stratified by the treatment of heroin use disorder. The infectious disease branches followed the heroin use disorder treatment branches in a similar arrangement (Fig 1). Certain other inputs, such as percentage of users treated for a health condition (e.g. heroin use disorder, chronic infectious diseases, etc.) and associated costs, overdose treatment and costs, neonatal abstinence syndrome (NAS) treatment and costs, lost productivity costs, and cost of heroin to the user, were not explicitly shown as branches, and relevant costs were instead applied to the model as a weighted average per branch; this process is described in more detail later in the methods section. The chronic infectious diseases and NAS along with their treatment costs were chosen for inclusion in the model due to their heightened risk in heroin users [14, 15], association with each other [16], and/or high cost of treatment.[17]

Mutually exclusive and exhaustive events occurred by literature-identified probabilities at each branching point shown in Fig 1. The model has been simplified; each symbol represents an identically structured clone of branches. The ordering of model branches from left to right was not reflective of the chronological order of events, but instead the conditional prevalence rates of combinations of states (e.g. an incarcerated individual having HBV as well if he/she is HCV-positive) in that pathway. Each pathway represents a mutually exclusive combination of health and incarceration states included in the model; further assumptions of the model are provided in S1 List.

### Model inputs

Medline, Google Scholar (each with results limited to English), and US governmental agency websites were searched to identify model inputs. The model inputs included: number of heroin users in the general and prison populations (used to estimate proportion of incarcerated users); prevalence rates, treatment costs, and treatment rates for heroin use disorder, infectious diseases, and NAS associated with heroin use; cost of incarceration; productivity loss due to heroin use or incarceration; crime costs associated with heroin use; cost of heroin to users; mortality due to heroin overdose; and probability and cost of heroin-related overdose (Table 1).

**Table 1:** Model inputs with years of publication for references.

Variable	Non-incarcerated heroin users		Incarcerated heroin users	
	Model Input [ref]	Year of citation publication	Model input [ref]	Year of citation publication
Number of heroin users	808,000[18]	2015	200,000[9]	2007
Probability of use treatment	0.110[19]	2015	0.141[20]	2007
Cost of heroin use disorder treatment	$9,187.08[21]	2011	$9,187.08[21]	2011
Probability of HIV infection	0.070[22]	2015	0.310[23]	2015
Probability of HIV treatment	0.305[24]	2013	0.333[25]	2010
Cost of HIV treatment	$23,681.71[26]	2010	$23,681.71[26]	2010
Probability of having HCV given HIV+	0.800[27]	2014	0.700[28]	2005
Probability of having HCV given HIV-	0.769[29]	1996	0.657[30]	2005
Probability of HCV treatment	0.160[31]	2008	0.160[31]	2008
Cost of HCV treatment	$81,633.51[32]	2016	$81,633.51[32]	2016
Probability of HBV given only HIV+	0.071[33]	2003	0.082[34]	2009
Probability of HBV given only HCV+	0.081[35]	2010	0.013[34]	2009
Probability of HBV given HIV+ and HCV+	0.081[35]	2010	0.082[34]	2009
Probability of HBV given HIV- and HCV-	0.035[36]	2011	0.082[34]	2009
Probability of HBV treatment	0.140[37]	2014	0.140[37]	2014
Cost of HBV treatment	$28,817.62[38]	2004	$28,817.62[38]	2004
Probability of TB given HIV+	0.160[39]	2008	0.160[39]	2008
Probability of TB given no chronic infectious diseases	0.390[39]	2008	0.390[39]	2008
Probability of TB treatment	0.050[40, 41]	2009,2009	0.050[40, 41]	2009,2009
Cost of TB treatment	$545.15[42]	2009	$545.15[42]	2009
Probability of overdose death while on use treatment	0.0023[43]	2015	0.0023[43]	2015
Probability of overdose death while not on use treatment	0.0080[43]	2015	0.0080[43]	2015
Probability of experiencing overdose and living	0.339[44, 45]	2013,2009	0.339[44, 45]	2013, 2009
Cost to treat each overdose	$3369.71[44]	2013	$3369.71[44]	2013
Cost of crime committed by each user	$6795.77[21]	2011	N/A	N/A
Cost of incarceration	N/A	N/A	$30,656.20[46]	2015
Lost productivity by non-incarcerated users who live^1^	$4,910.53[21, 47]	2011, 2014	N/A	2011, 2014
Lost productivity by non-incarcerated users who die ^1^	$28,885.46[21, 47]	2011, 2014	N/A	N/A
Lost productivity by incarcerated users^1^	N/A	N/A	$28,885.46[21, 47]	2011, 2014
Probability of Neonatal Abstinence Syndrome treatment	0.0148[48]	2015	0.0148[48]	2015
Cost of Neonatal Abstinence Syndrome treatment	$68,856.48[49]	2015	$68,856.48[49]	2015
Cost of Heroin to User^2^	$19,004.32[50]	2014	N/A	N/A

^1^The median salary was included in the model because an individual would be capable of such earnings if he/she did not have heroin use disorder; in effect, society loses these earnings as the individual is unable to work at the same capacity as a normal member of society

^2^The cost of heroin to the user is N/A for incarcerated users to reflect that although users may have access to heroin while incarcerated, they are unlikely to be using currency to obtain the heroin; thus, the cost of heroin to user is assumed as $0

Costs of treatment for heroin use disorder, overdose, NAS, and infectious diseases were assumed to be the same amongst the incarcerated and non-incarcerated populations; these treatment costs were weighted by the rates of treatment for the condition to calculate an expected treatment cost for patients (not shown in Fig 1). Relevant drug overdose costs were also applied to each pertinent health state. For example, 33.9% of heroin users were projected to experience overdose and survive based on contemporary literature estimates.[44, 45] Only this proportion of living users was assumed to incur overdose treatment costs. A similar procedure was applied to NAS treatment costs. Patients were assumed to have remained in the same health and/or incarceration state (e.g. incarcerated with HCV) for the entire year. Productivity losses were based on the median national wage for 2014 (converted to 2015 dollars) in the United States[47], which was the last year of data available at the time of this study. In accordance with a recent US Department of Justice Report, non-incarcerated, living patients lost 17% of their wages.[21] All costs were converted to 2015 dollars using the Consumer Price Index (CPI); full cost updates are shown in S1 Table. Only deaths directly attributed to heroin overdoses were included as part of the model, i.e. deaths due to infectious diseases were not included, because the rate of such deaths was substantially lower in comparison to the rate of overdose deaths; overdose death risks were assumed to be different between those who were on and off heroin use treatment.[43]

When several data sources were available for a model input, the following criteria were applied to guide selection for the base case: comparability of study population to US heroin users, study sample size, strength of study design, duration and recentness of study. When estimates were not available, the model was populated using similar data (S1 List) or inputs were derived using known information (S1 Calculations).

### Sensitivity analyses

One-way sensitivity analyses were conducted for all inputs with multiple values found in literature by varying the values across ranges found in the literature; one-way sensitivity analyses were also conducted for specific inputs related to certain assumptions as necessary, even if no range of inputs was found in the literature. If variation in the model input caused the estimated cost of heroin use disorder to change by 10% or more, the estimate was considered to be sensitive to the model input. Monte-Carlo simulation was conducted with 100,000 iterations using all inputs which affected the estimated cost by at least 1% on one-way sensitivity analyses.

## Results

### Base case results

**Fig 2:**
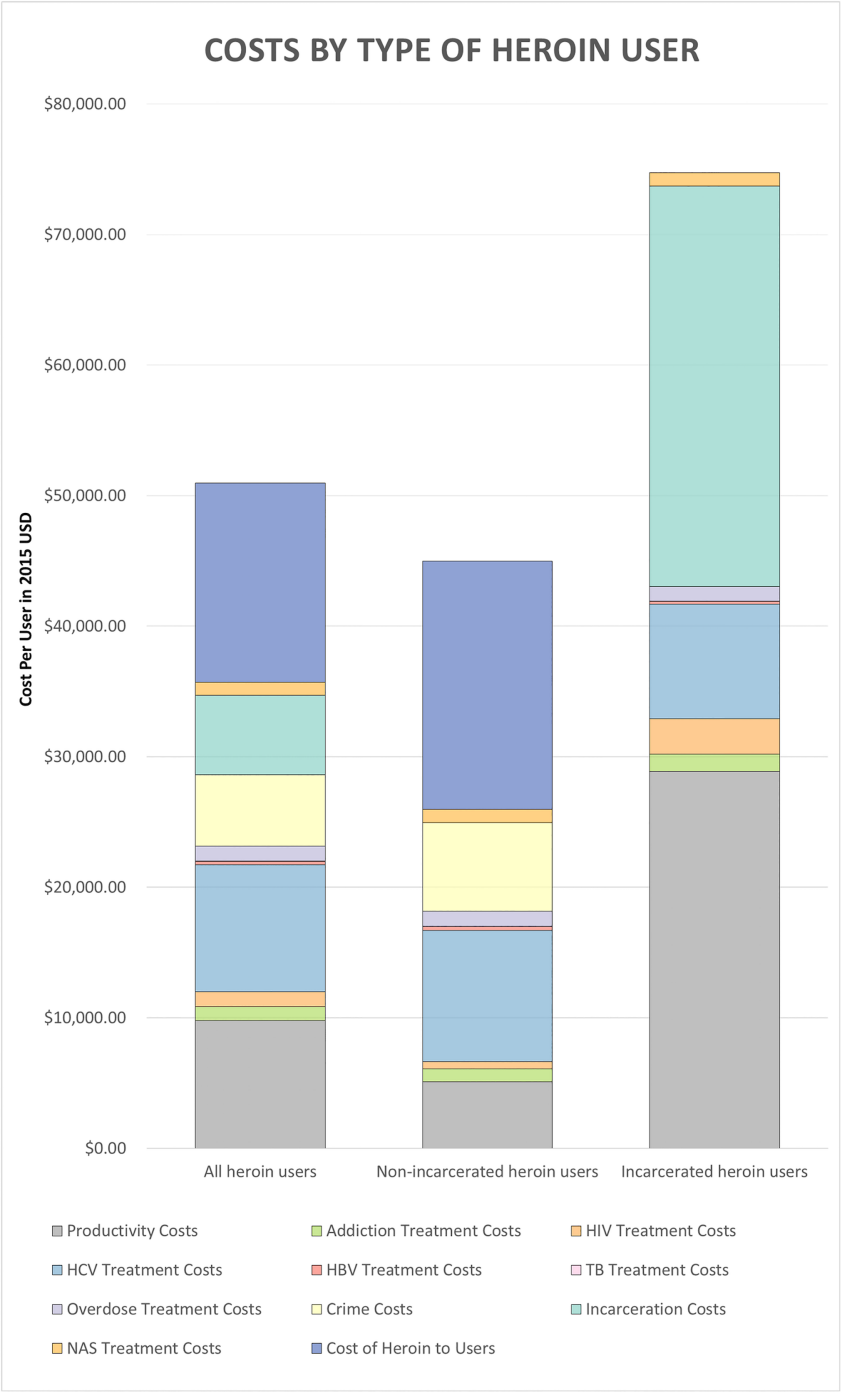
Costs per user by type of heroin user. Exact values contained in S2 Table.

The estimated total cost of heroin use disorder in the United States was $51.2 billion in 2015 US dollars, with an average of $50,799 per heroin user (Fig 2; S2 Table). Among the overall population with heroin use disorder, the productivity loss per user ($9,809; 19.3%), HCV treatment ($9,740; 19.2%), crime ($5,447; 10.7%), incarceration ($6,083; 12.0%), and cost of heroin to the user ($15,234; 30.0%) constituted over 90% of the costs of heroin use disorder. The mean cost associated with a non-incarcerated heroin user was $44,950; for each non-incarcerated user, productivity losses amounted to $5,087 (11.3%); HCV treatment cost $10,073 (22.4%); crime costs were $5,491 (15.1%); and heroin cost to users were $19,004 (42.3%). Each incarcerated heroin user cost society $74,428, mostly driven by productivity loss ($28,885; 38.8%), incarceration costs ($30,656; 41.2%), and HCV treatment costs ($8,755; 11.8%).

### Sensitivity analyses

**Fig 3:**
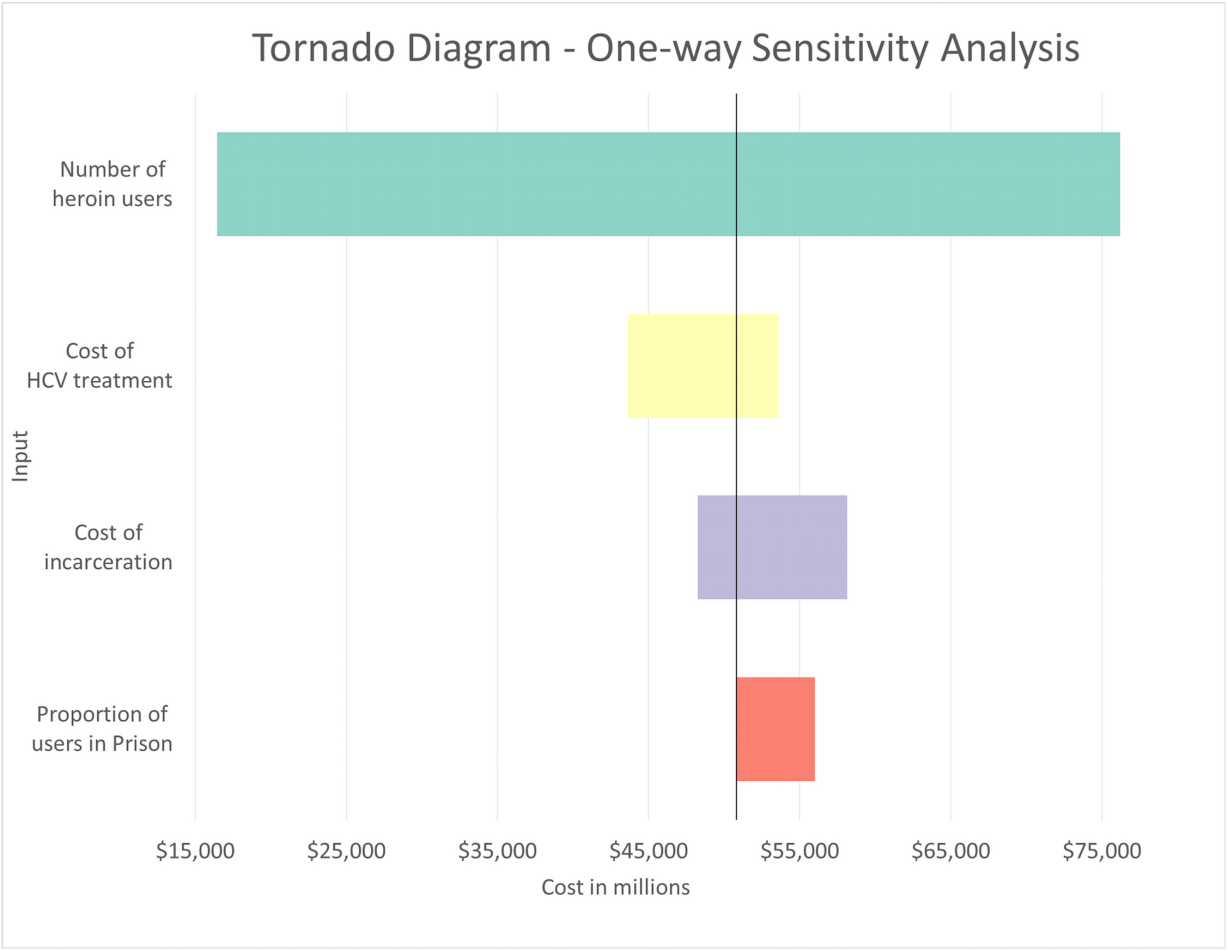
Tornado diagram one-way sensitivity analyses. Full values contained in S3 Table.

In one-way sensitivity analyses, when the number of individuals with heroin use disorder was varied between 324,000 (people currently using heroin)[18] and 1.5 million (chronic users–people who used heroin for 4 or more days in the last month)[50] based on possible different definitions of heroin use disorder, the total cost ranged from $16.5 billion to $76.2 billion, or 32.1% to 148.8% of base case cost (Table 2 and Fig 3). Using a range of HCV treatment costs ranging from the past standard of care ($18,977)[51] to the current standard of care for patients with cirrhosis ($101,380)[32], the overall estimated cost was calculated to be between $43.6 billion and $53.2 billion (85.2% to 104.7% of base case). The model was sensitive to the cost of incarceration when it was varied between $15,873 and $65,300[52]—results ranged from 94.2% to 113.5% of base case value. The estimate was also sensitive to the cost of incarceration. When the proportion of users in prison was varied between 19.8%[9, 18] and 36%[9] (base case 19.8%), the overall estimate increased to $56.0 billion (109.4% of base case). Full one-way sensitivity results are found in S3 Table.

**Table 2:** One-way sensitivity analyses results, in descending order of impact on results.

Variable	Base Value [ref]	Low Input [ref]	High Input [ref]	Range of total heroin cost (in millions)	
Number individuals with heroin use disorder	1,008,000[9, 18]	324,000[18]	1,500,000[50]	$16,458.92	$76,198.71
Cost of HCV treatment	$81,633.51[32]	$18,977[51]	$101,380[32]	$43,614.85	$53,597.77
Cost of incarceration	$30,656.20[46]	$15,872.80[53]	$65,299.89[53]	$48,248.85	$58,134.27
Proportion of users in prison*	0.198[9, 18]	0.198[9, 18]	0.36[9]	$51,205.15	$56,006.82

*Marginally significant; 9.4% change from baseline

Using 100,000 samples in a Monte Carlo simulation with the top 9 model inputs that the model results are most sensitive to in one-way sensitivity analyses, the mean per-user cost was $57,115.76 with a standard deviation (SD) of $5,619.45 (95% confidence interval [CI] $47,043.49 to $68,620.78); the cost for the overall population was $52.1 billion (SD $20.1 billion; 95% CI $19.7 billion to $89.3 billion).

## Discussion

The US societal cost of heroin use disorder was estimated to be $51.2 billion for 1,008,000 heroin users (an average of $50,799 per user) in 2015 US dollars. To put the overall annual economic in context, we present the annual societal costs of some other chronic illnesses. For example, chronic obstructive pulmonary disease (COPD) was estimated to cost $2,567 per patient ($38.50 billion in 2015 dollars for 15 million patients), and diabetes was estimated to cost $11,148 per patient ($248.59 billion in 2015 dollars for 22.3 million patients).[54, 55] Our study helps to contextualize the cost per individual with heroin use disorder to society, which is substantially higher than the per-patient cost for conditions such as COPD and diabetes.

A previous study by Mark et al. estimated the societal cost of heroin addiction (the previous term for heroin use disorder) to be $21.9 billion in 1996 dollars[56] (33.1 billion in 2015 dollars). The Mark et al. study estimated a per-user cost of $55,167 in 2015 dollars, whereas our contemporary estimate from the current study was $50,799. The substantial difference in total cost of heroin addiction/user disorder, 51.2 billion versus 33.1 billion, despite similar per-user cost across studies, is likely the different population sizes included in each study; 1,008,000 was used as the population size in this study as compared to the 600,000 used in the Mark et al. study.[56]

Although both the Mark study and the present study calculated 1-year societal costs of prevalent heroin addiction/use disorder, the methods, scope, and population differed between studies. When a user died, Mark et al. quantified productivity loss for the remainder of the patient’s working life, defined as the number of years between age at death and average retirement age.[56] This approach is typically only used in incidence-based studies[21] and overestimates the productivity loss contribution to the overall cost in a prevalence cost of illness study. Thus, the Mark et al. study inappropriately employed both prevalence-based (i.e. annual) and incidence-based (i.e. lifetime) cost of illness methods. In the present study, only the productivity loss for 1 year was included in the overall cost calculation following user death or incarceration to maintain consistency with the typical prevalence cost-of-illness study methods.[57] If we used comparable methods, our estimates would have been substantially higher. Productivity loss accounted for 52.6% of the overall costs in the Mark et al. study ($29,018 in 2015 dollars)[56] as compared to the 19.3% of the overall costs in this study ($9,809). Finally, the demographics of the heroin-using population have changed since the 1990s (with a greater proportion of women and larger proportion who are White), and model inputs for disease, crime, and others for cost calculation likely changed as well.[58, 59] Thus, the populations studied in the Mark study and the present study are not directly comparable.

There are several limitations to estimating the societal cost of heroin use disorder. The nature of heroin use disorder is complex; nearly all (96%) of heroin users abused at least 1 other substance (prescription opioids, marijuana, alcohol).[2] Additionally, some evidence suggests that many patients transition from prescription opioids to heroin,[60] while other evidence suggests prescription opioid abuse and heroin use disorder are separate phenomena.[61] Thus, the degrees of overlap between the abuses of various substances are difficult to determine without making assumptions that are difficult to verify. We chose to report the data on heroin use disorder as is as not to introduce additional error by adding more assumptions.

Although the model was developed while considering both available data and clinical knowledge, some conditional probabilities of disease prevalence were not found in the literature; in the absence of these figures, certain assumptions were made (S1 List). For example, HIV was assumed to be the most important risk factor for TB [16], thus the prevalence of TB in all HIV-positive patient groups was assumed to be the same, regardless of what other chronic disease(s) were present in the group. Intravenous drug use is a shared risk factor between all the chronic infectious diseases;[56, 62] thus, due to the interdependent nature of the diseases, such an assumption of disease prevalence rates undervalues the proportions of users with co-infection and leads to underestimation of the costs of heroin use disorder. Acute infections (e.g. pneumonia, skin and soft tissue infections) associated with heroin use were also not included in this study due to the difficulty of obtaining an accurate cross-section estimate of such illnesses, again underscoring the conservative nature of our estimate.

The downstream externalities of heroin use disorder, treatment of chronic infection(s), and treatment of heroin use disorder were not captured in this model. Treatment of these patients for their disease(s) may drastically reduce their chances of transmitting the viruses to others [63, 64], and treating patients’ heroin use has been shown to decrease rates of new infections.[65] If IV drug users with heroin use disorder were studied through their entire drug use lifetimes with yearly addition of incident users into the model, the effects of such interventions could be better quantified. Further, public health initiatives such as needle-exchange programs and their resulting impacts were not included in this analysis. Additional research is needed on the impact of these externalities and public health programs.

The generalizability and reliability of estimates for some model inputs were limited, as the source studies were often conducted in a single location with a small sample size. It is challenging to conduct studies on drug using populations (e.g. to measure the prevalence of infectious diseases), particularly imprisoned heroin users.[66, 67] For inputs for which several published studies exist, prevalence rates often varied widely for both incarcerated and non-incarcerated populations. To explore the impact of these different estimates on the results, literature-based input ranges were evaluated across the input spectrum to explore their impact on the results; the results were robust to most inputs (S3 Table). A major unknown factor was whether incarcerated and non-incarcerated patients with heroin use disorder were treated at similar frequencies for health conditions and/or addiction treatment; this uncertainty was evaluated in one-way sensitivity analysis by varying the ratio of treatment of incarcerated individuals to non-incarcerated individuals by 25% (0.75 to 1.25). The estimate was found to be robust to this variation as shown in S3 Table. Overall, the model results were also robust to probabilistic sensitivity analyses for per-user cost; the Monte Carlo simulation demonstrated a wide range for the total heroin user disorder cost to society, further emphasizing the uncertainty in the estimated number of individuals with heroin use disorder in the US.

A major strength of this study was the consistent application of a prevalence-based approach to estimating cost of illness and careful choice of inputs based on stated criteria for the population of interest. Additionally, the model-guided calculation of cost of heroin use disorder in this study allowed for the detailed determination of productivity loss and treatment costs by health state. Mark et al. chose to sum relevant costs and applied a different framework than the approach of this study and could then include a wider scope of costs.[56] Although the structured model design narrowed the scope of this study (e.g. did not allow for inclusion of social welfare costs), the model did allow for a more precise estimate by considering information specific to each health state, such as the distribution of patients with infectious disease(s) and the proportion of treated patients.

Sources vary in the prevalence of heroin use disorder, depending on the definition by the researcher; 200,000 incarcerated heroin users,[9] 324,000 current heroin users (previously referred to as physically dependent on heroin),[18] 808,000 past-year heroin users (previously referred to as heroin use disorder)[18], and 1.5 million total users who used heroin at least 4 times in the past month[50] have all been published in the literature. To be clear, in 2015, the National Survey on Drug Use and Health (NSDUH) survey, the yearly survey from which we draw our data, changed its wordings from heroin use disorder to past-year heroin use and from heroin physical dependence to current use.[68] The figures between years are assumed to be comparable to their former wording by Substance Abuse and Mental Health Services Administration (SAMHSA), the administrator of the NSDUH; thus, past-year heroin use is synonymous with heroin use disorder (or addiction, the previously accepted term) and current heroin use is synonymous with heroin physical dependence. The discrepancy in estimates may be attributed to the different definitions used by various agencies, e.g. past-year user, use of heroin for 4 or more days per month, etc., but a precise estimate of heroin use disorder may be nearly impossible due to issues previously described.[66] Similarly, various studies on disease prevalence, treatment costs, etc. operationalized heroin addiction/heroin use disorder differently or did not report the exact definition used; out of necessity, the reported estimates were used in our study even if the study definition was not identical to that used in our study.

In choosing a base-case value for the number of individuals with heroin use disorder, we expected past-year users/users with heroin use disorder and current/physically dependent users to have similar societal costs, and exclusion of users who were not heroin dependent nor actively currently using heroin from the study would severely underestimate the cost of heroin use disorder. Additionally, defining chronic heroin use as those who used at least 4 times in the past month[66] may be over-estimating the population heavily impacted by heroin use disorder. Thus, heroin use disorder was operationalized as heroin use within the last year (808,000 adult non-incarcerated users [18] in 2015). Minors with a heroin use disorder (21,000 individuals aged 12 to 17)[18] were excluded from the study because their cost to society may be systematically different from the adult population. The omission of past-year heroin users less than 18 years of age likely biased the estimate downwards.

The range of 324,000 (people currently using heroin)[18] to 1.5 million (chronic users of heroin[50]) was tested to evaluate the change in results with varied definitions of heroin use disorder (Fig 2; S3 Table). In the base case, the non-incarcerated population was assumed to not overlap with the incarcerated population; by varying both the number of heroin users and the portion of incarcerated heroin users (19.8% to 36%[9]) individually, the robustness of this assumption was tested. The estimate was sensitive to the number of users and marginally sensitive to the portion of incarcerated users.

The total cost was also sensitive to the cost of HCV treatment. Because the cost of HCV treatment has recently increased dramatically due to the introduction of several novel HCV treatment options,[59] this aspect of model sensitivity is especially relevant. As compared to the previous standard of care (ribavirin and pegylated interferon), these new options allow the HCV treatment regimen to be entirely oral, contain a more favorable side effect profile, have increased cure rates, and require shorter treatment duration.[69] However, these treatments can range in cost from $83,000 to over $150,000 for a course of therapy. Although the medications have been shown to be cost-effective, concerns about the affordability may prevent complete adoption of these medications as first-line treatment.[69, 70] As demonstrated in our sensitivity analysis, if the cost of current HCV treatment remained the same price as that of pegylated interferon and ribavirin (18,977),[51] the total cost of heroin use disorder would have decreased 14.8% to $43.6 billion.

Without meaningful public health efforts, the number of heroin users is likely to continue to grow; the downstream effects of heroin use, such as the spread of infectious diseases[56, 62] and increased incarceration due to actions associated with heroin use,[56] compounded by their associated costs would continue to increase the societal burden of heroin use disorder. The results of the sensitivity analyses suggest that implementation of healthcare policy which targets reduction in the number of heroin users and decreases in the cost of HCV treatment may help to mitigate healthcare costs and productivity losses associated with heroin use. The high cost of incarcerated heroin users compared to non-incarcerated users suggests that criminal laws aimed to reduce the number or proportion of incarcerated heroin users may help to control costs. In March 2016, the Obama administration announced plans to focus on treatment of heroin use disorder/addiction rather than incarceration so that former users may return to full productivity.[71, 72] Increased treatment for heroin use disorder may also reduce the users’ heroin expenses; Substance Abuse and Mental Health Services Association (SAMHSA) implemented new legislation in August 2016 to increase the buprenorphine treatment limit per qualified doctor from 100 patients to 275. This policy may be especially helpful in rural areas where there is a shortage of buprenorphine prescribers and where many heroin users reside. Although these policies should be beneficial in reducing both the humanistic and economic outcomes of heroin use disorder in principle, the effect of these policies on societal cost of heroin use disorder remains to be seen.

Despite the limitations of this study, many of which are inherent to cost of illness studies, the study provides important evidence to inform policy on combating the heroin epidemic. The societal cost of heroin use disorder has not been characterized since 1996, and the results of this study use the most recently available data and trends to provide a cost estimate of the burden of heroin use disorder to society in the United States. Even with a comparatively narrow perspective, heroin use disorder exacts a tremendous cost to society at $50,799 per user. Possible targets for reduction of the societal cost of heroin use disorder such as reducing the overall number of heroin users, reducing the proportion of users who are incarcerated, and others were also identified. Downstream effects of heroin use such as newly acquired infections due to needle sharing or high-risk sexual behaviors and the effects of heroin use disorder and chronic infectious disease treatments were not captured in this study; additional research is needed for better characterization of these outcomes.

## Supporting information

S1 Calculations Calculations for inputs derived from published sources. (DOCX)

S1 List Model assumptions. (DOCX)

S1 Table Conversion of costs found in literature to 2015 costs using consumer price index. (DOCX)

S2 Table Results: Costs by type; shown by heroin user classification (population total and by incarceration status). (DOCX)

S3 Table Full one-way sensitivity analyses results. (DOCX)

## References

[1] Jones CM. Heroin use and heroin use risk behaviors among nonmedical users of prescription opioid pain relievers—United States, 2002–2004 and 2008–2010. Drug Alcohol Depend. 2013;132(1–2):95–100. doi: 10.1016/j.drugalcdep.2013.01.007 23410617 10.1016/j.drugalcdep.2013.01.007

[2] Jones CM, Logan J, Gladden RM, Bohm MK. Vital Signs: Demographic and Substance Use Trends Among Heroin Users—United States, 2002–2013. MMWR Morb Mortal Wkly Rep. 2015;64(26):719–25. 26158353 PMC4584844

[3] Rudd RA, Aleshire N, Zibbell JE, Gladden RM. Increases in Drug and Opioid Overdose Deaths—United States, 2000–2014. MMWR Morb Mortal Wkly Rep. 2016;64(50–51):1378–82. doi: 10.15585/mmwr.mm6450a3 26720857 10.15585/mmwr.mm6450a3

[4] Centers for Disease Control and Prevention. Today’s Heroin Epidemic Infographics [updated July 7, 2015; cited 2016 November 4]. Available from: http://www.cdc.gov/vitalsigns/heroin/infographic.html - graphic.

[5] National Drug Threat Assessment—Impact on Society 2010 [cited 2016 July 1]. Available from: https://www.justice.gov/archive/ndic/pubs38/38661/drugImpact.htm.

[6] National Drug Threat Assessment US Department of Justice National Drug Intelligence Center; 2011 [cited 2016 January 29]. Available from: http://www.justice.gov/archive/ndic/pubs44/44849/44849p.pdf.

[7] Tetrault JM, Fiellin DA. Current and potential pharmacological treatment options for maintenance therapy in opioid-dependent individuals. Drugs. 2012;72(2):217–28. PubMed Central PMCID: PMCPMC3701303. doi: 10.2165/11597520-000000000-00000 22235870 10.2165/11597520-000000000-00000 PMC3701303

[8] Teesson M, Marel C, Darke S, Ross J, Slade T, Burns L, et al Long-term mortality, remission, criminality and psychiatric comorbidity of heroin dependence: 11-year findings from the Australian Treatment Outcome Study. Addiction. 2015;110(6):986–93. doi: 10.1111/add.12860 25619110 10.1111/add.12860

[9] Boutwell AE, Nijhawan A, Zaller N, Rich JD. Arrested on heroin: a national opportunity. J Opioid Manag. 2007;3(6):328–32. 18290584 10.5055/jom.2007.0021

[10] Elliott JC, Hasin DS, Stohl M, Des Jarlais DC. HIV, Hepatitis C, and Abstinence from Alcohol Among Injection and Non-injection Drug Users. AIDS Behav. 2015. 10.1007/s10461-015-1113-z PMC5047517 26080690

[11] Factor SH, Sackoff JE, Raj-Singh S, Wu Y, Monserrate J, Munsiff S, et al Street-outreach improves detection but not referral for drug users with latent tuberculosis, New York City. Subst Use Misuse. 2011;46(14):1711–5. doi: 10.3109/10826084.2011.615562 21943282 10.3109/10826084.2011.615562

[12] Weaver T, Metrebian N, Hellier J, Pilling S, Charles V, Little N, et al Use of contingency management incentives to improve completion of hepatitis B vaccination in people undergoing treatment for heroin dependence: a cluster randomised trial. Lancet. 2014;384(9938):153–63. doi: 10.1016/S0140-6736(14)60196-3 24725468 10.1016/S0140-6736(14)60196-3

[13] Owusu-Edusei K Jr., Chesson HW, Gift TL, Tao G, Mahajan R, Ocfemia MC, et al The estimated direct medical cost of selected sexually transmitted infections in the United States, 2008. Sex Transm Dis. 2013;40(3):197–201. doi: 10.1097/OLQ.0b013e318285c6d2 23403600 10.1097/OLQ.0b013e318285c6d2

[14] National Institute on Drug Abuse. Why does heroin use create special risk for contracting HIV/AIDS and hepatitis B and C? [updated November 2014; cited 2017 March 23]. Available from: https://www.drugabuse.gov/publications/research-reports/heroin/why-are-heroin-users-special-risk-contracting-hivaids-hepatitis-b-c.

[15] National Institute on Drug Abuse. How does heroin use affect pregnant women? [updated November 2014; cited 2017 March 23]. Available from: https://www.drugabuse.gov/publications/research-reports/heroin/how-does-heroin-abuse-affect-pregnant-women.

[16] Centers for Disease Control and Prevention. TB Risk Factors [updated March 18, 2016; cited 2017 March 23]. Available from: https://www.cdc.gov/tb/topic/basics/risk.htm.

[17] Patrick SW, Schumacher RE, Benneyworth BD, Krans EE, McAllister JM, Davis MM. Neonatal abstinence syndrome and associated health care expenditures: United States, 2000–2009. JAMA. 2012;307(18):1934–40. doi: 10.1001/jama.2012.3951 22546608 10.1001/jama.2012.3951

[18] Center for Behavioral Health Statistics and Quality. Key substance use and mental health indicators in the United States: Results from the 2015 National Survey on Drug Use and Health (HHS Publication No. SMA 16–4984, NSDUH Series H-51). 2016 [cited 2016 October 15]. Available from: http://www.samhsa.gov/data/.

[19] National Institute on Drug Abuse. DrugFacts: Nationwide Trends [updated June 2015; cited 2016 Feburary 1]. Available from: http://www.drugabuse.gov/publications/drugfacts/nationwide-trends.

[20] Mumola CJK, J.C. Drug Use and Dependence, State and Federal Prisoners, 2004 2006 [cited 2016 July 29]. Available from: http://www.bjs.gov/content/pub/pdf/dudsfp04.pdf.

[21] US Department of Justice and National Drug Intelligence Center. The Economic Impact of Illicit Drug Use on American Society 2011 [cited 2016 January 29]. Available from: http://www.justice.gov/archive/ndic/pubs44/44731/44731p.pdf.

[22] Spiller MW, Broz D, Wejnert C, Nerlander L, Paz-Bailey G, Centers for Disease C, et al HIV infection and HIV-associated behaviors among persons who inject drugs—20 cities, United States, 2012. MMWR Morb Mortal Wkly Rep. 2015;64(10):270–5. 25789742 PMC4584803

[23] Genberg BL, Astemborski J, Vlahov D, Kirk GD, Mehta SH. Incarceration and injection drug use in Baltimore, Maryland. Addiction. 2015;110(7):1152–9. PubMed Central PMCID: PMCPMC4478154. doi: 10.1111/add.12938 25845621 10.1111/add.12938 PMC4478154

[24] Westergaard RP, Hess T, Astemborski J, Mehta SH, Kirk GD. Longitudinal changes in engagement in care and viral suppression for HIV-infected injection drug users. AIDS. 2013;27(16):2559–66. PubMed Central PMCID: PMCPMC3795966. doi: 10.1097/QAD.0b013e328363bff2 23770493 10.1097/QAD.0b013e328363bff2 PMC3795966

[25] Wakeman SE, Rich JD. HIV treatment in US prisons. HIV Ther. 2010;4(4):505–10. PubMed Central PMCID: PMCPMC2953806. 20953349 10.2217/hiv.10.35 PMC2953806

[26] Gebo KA, Fleishman JA, Conviser R, Hellinger J, Hellinger FJ, Josephs JS, et al Contemporary costs of HIV healthcare in the HAART era. AIDS. 2010;24(17):2705–15. PubMed Central PMCID: PMCPMC3551268. doi: 10.1097/QAD.0b013e32833f3c14 20859193 10.1097/QAD.0b013e32833f3c14 PMC3551268

[27] Center for Disease Control. HIV and Viral Hepatitis Fact Sheet 2014 [cited 2016 February 1]. Available from: http://www.cdc.gov/hiv/pdf/library_factsheets_hiv_and_viral_hepatitis.pdf.

[28] Weinbaum CM, Sabin KM, Santibanez SS. Hepatitis B, hepatitis C, and HIV in correctional populations: a review of epidemiology and prevention. AIDS. 2005;19 Suppl 3:S41–6. 16251827 10.1097/01.aids.0000192069.95819.aa

[29] Garfein RS, Vlahov D, Galai N, Doherty MC, Nelson KE. Viral infections in short-term injection drug users: the prevalence of the hepatitis C, hepatitis B, human immunodeficiency, and human T-lymphotropic viruses. Am J Public Health. 1996;86(5):655–61. PubMed Central PMCID: PMCPMC1380472. 8629715 10.2105/ajph.86.5.655 PMC1380472

[30] Fox RK, Currie SL, Evans J, Wright TL, Tobler L, Phelps B, et al Hepatitis C virus infection among prisoners in the California state correctional system. Clin Infect Dis. 2005;41(2):177–86. doi: 10.1086/430913 15983913 10.1086/430913

[31] Grebely J, Genoway KA, Raffa JD, Dhadwal G, Rajan T, Showler G, et al Barriers associated with the treatment of hepatitis C virus infection among illicit drug users. Drug Alcohol Depend. 2008;93(1–2):141–7. doi: 10.1016/j.drugalcdep.2007.09.008 17997050 10.1016/j.drugalcdep.2007.09.008

[32] Younossi ZMP, Haesuk Gordon, Stuart C.; Ferguson John R.; Ahmed Aijaz; Dieterich Douglas; Saab Sammy. Real-World Outcomes of Ledipasvir/Sofosbuvir in Treatment-Naïve Patients With Hepatitis C Am J Manag Care. 2016. 27266950

[33] Kellerman SE, Hanson DL, McNaghten AD, Fleming PL. Prevalence of chronic hepatitis B and incidence of acute hepatitis B infection in human immunodeficiency virus-infected subjects. J Infect Dis. 2003;188(4):571–7. doi: 10.1086/377135 12898445 10.1086/377135

[34] Hennessey KA, Kim AA, Griffin V, Collins NT, Weinbaum CM, Sabin K. Prevalence of infection with hepatitis B and C viruses and co-infection with HIV in three jails: a case for viral hepatitis prevention in jails in the United States. J Urban Health. 2009;86(1):93–105. PubMed Central PMCID: PMCPMC2629523. doi: 10.1007/s11524-008-9305-8 18622707 10.1007/s11524-008-9305-8 PMC2629523

[35] Bini EJ, Perumalswami PV. Hepatitis B virus infection among American patients with chronic hepatitis C virus infection: prevalence, racial/ethnic differences, and viral interactions. Hepatology. 2010;51(3):759–66. doi: 10.1002/hep.23461 20140950 10.1002/hep.23461

[36] Nelson PK, Mathers BM, Cowie B, Hagan H, Des Jarlais D, Horyniak D, et al Global epidemiology of hepatitis B and hepatitis C in people who inject drugs: results of systematic reviews. Lancet. 2011;378(9791):571–83. PubMed Central PMCID: PMCPMC3285467. doi: 10.1016/S0140-6736(11)61097-0 21802134 10.1016/S0140-6736(11)61097-0 PMC3285467

[37] Sarkar M, Shvachko VA, Ready JB, Pauly MP, Terrault NA, Peters MG, et al Characteristics and management of patients with chronic hepatitis B in an integrated care setting. Dig Dis Sci. 2014;59(9):2100–8. PubMed Central PMCID: PMCPMC4149592. doi: 10.1007/s10620-014-3142-2 24728968 10.1007/s10620-014-3142-2 PMC4149592

[38] Lavanchy D. Hepatitis B virus epidemiology, disease burden, treatment, and current and emerging prevention and control measures. J Viral Hepat. 2004;11(2):97–107. 14996343 10.1046/j.1365-2893.2003.00487.x

[39] Golub JE, Astemborski J, Ahmed M, Cronin W, Mehta SH, Kirk GD, et al Long-term effectiveness of diagnosing and treating latent tuberculosis infection in a cohort of HIV-infected and at-risk injection drug users. J Acquir Immune Defic Syndr. 2008;49(5):532–7. PubMed Central PMCID: PMCPMC2637943. doi: 10.1097/QAI.0b013e31818d5c1c 18989223 10.1097/QAI.0b013e31818d5c1c PMC2637943

[40] Hauck FR, Neese BH, Panchal AS, El-Amin W. Identification and management of latent tuberculosis infection. Am Fam Physician. 2009;79(10):879–86. 19496388

[41] Deiss RG, Rodwell TC, Garfein RS. Tuberculosis and illicit drug use: review and update. Clin Infect Dis. 2009;48(1):72–82. PubMed Central PMCID: PMCPMC3110742. doi: 10.1086/594126 19046064 10.1086/594126 PMC3110742

[42] Holland DP, Sanders GD, Hamilton CD, Stout JE. Costs and cost-effectiveness of four treatment regimens for latent tuberculosis infection. Am J Respir Crit Care Med. 2009;179(11):1055–60. PubMed Central PMCID: PMCPMC2689913. doi: 10.1164/rccm.200901-0153OC 19299495 10.1164/rccm.200901-0153OC PMC2689913

[43] Evans E, Li L, Min J, Huang D, Urada D, Liu L, et al Mortality among individuals accessing pharmacological treatment for opioid dependence in California, 2006–10. Addiction. 2015;110(6):996–1005. PubMed Central PMCID: PMCPMC4452110. doi: 10.1111/add.12863 25644938 10.1111/add.12863 PMC4452110

[44] Inocencio TJ, Carroll NV, Read EJ, Holdford DA. The economic burden of opioid-related poisoning in the United States. Pain Med. 2013;14(10):1534–47. doi: 10.1111/pme.12183 23841538 10.1111/pme.12183

[45] Substance Abuse and Mental Health Services Administration. Results from the 2009 National Survey on Drug Use and Health: Volume I. Summary of National Findings 2009 [cited 2016 November 10]. Available from: http://archive.samhsa.gov/data/NSDUH/2k9NSDUH/2k9Results.htm.

[46] Samuels J, CE. Annual Determination of Average Cost of Incarceration 2015 [cited 2016 July 27]. Available from: https://www.federalregister.gov/articles/2015/03/09/2015-05437/annual-determination-of-average-cost-of-incarceration.

[47] Social Security Administration. Measures Of Central Tendency For Wage Data—Average and Median Amounts of Net Compensation [cited 2016 July 18]. Available from: 7s://www.ssa.gov/oact/cola/central.html.

[48] Center for Behavioral Health Statistics and Quality Substance Abuse and Mental Health Services Administration. Results from the 2015 National Survey on Drug Use and Health: Detailed Tables [cited 2016 November 2]. Available from: http://www.samhsa.gov/data/sites/default/files/NSDUH-DetTabs-2015/NSDUH-DetTabs-2015/NSDUH-DetTabs-2015.pdf.

[49] Patrick SW, Davis MM, Lehmann CU, Cooper WO. Increasing incidence and geographic distribution of neonatal abstinence syndrome: United States 2009 to 2012. J Perinatol. 2015;35(8):650–5 PubMed Central PMCID: PMCPMC4520760. doi: 10.1038/jp.2015.36 25927272 10.1038/jp.2015.36 PMC4520760

[50] Kilmer B ES, Caulkins JP, et al. What America's Users Spend on Illicit Drugs: 2000 through 2010 2014 [cited 2016 July 29]. Available from: http://www.rand.org/pubs/research_reports/RR534.html.

[51] Solomon M, Bonafede M, Pan K, Wilson K, Beam C, Chakravarti P, et al Direct medical care costs among pegylated interferon plus ribavirin-treated and untreated chronic hepatitis C patients. Dig Dis Sci. 2011;56(10):3024–31 doi: 10.1007/s10620-011-1802-z 21717127 10.1007/s10620-011-1802-z

[52] Henrichson C, Delaney R. The Price of Prisons—What Incarceration Costs Taxpayers Center on Setencing and Corrections,; 2012 [updated July 20, 2012; cited 2017 April 3]. Available from: https://storage.googleapis.com/vera-web-assets/downloads/Publications/the-price-of-prisons-what-incarceration-costs-taxpayers/legacy_downloads/price-of-prisons-updated-version-021914.pdf.

[53] Henrichson CD, R. The Price of Prisons: What Incarceration Costs Taxpayers. New York: Vera Institute of Justice; 2012 [updated July 20, 2012; cited 2016 October 16]. Available from: http://archive.vera.org/sites/default/files/resources/downloads/price-of-prisons-updated-version-021914.pdf.

[54] Ford ES, Murphy LB, Khavjou O, Giles WH, Holt JB, Croft JB. Total and state-specific medical and absenteeism costs of COPD among adults aged ≥ 18 years in the United States for 2010 and projections through 2020. Chest. 2015;147(1):31–45. doi: 10.1378/chest.14-0972 25058738 10.1378/chest.14-0972

[55] American Diabetes Association. Economic costs of diabetes in the U.S. in 2012. Diabetes Care. 2013;36(4):1033–46. PubMed Central PMCID: PMCPMC3609540. doi: 10.2337/dc12-2625 23468086 10.2337/dc12-2625 PMC3609540

[56] Mark TL, Woody GE, Juday T, Kleber HD. The economic costs of heroin addiction in the United States. Drug Alcohol Depend. 2001;61(2):195–206. 11137285 10.1016/s0376-8716(00)00162-9

[57] Centers for Disease Control and Prevention—Division of Heart Disease and Stroke Prevention. Part II: Economic Impact Analysis. Cost of Illness: The Second of a Five-Part Series. Available from: http://www.cdc.gov/dhdsp/programs/spha/economic_evaluation/Module_II/index.htm.

[58] Cicero TJ, Ellis MS, Surratt HL, Kurtz SP. The changing face of heroin use in the United States: a retrospective analysis of the past 50 years. JAMA Psychiatry. 2014;71(7):821–6. doi: 10.1001/jamapsychiatry.2014.366 24871348 10.1001/jamapsychiatry.2014.366

[59] Thiagarajan P, Ryder SD. The hepatitis C revolution part 1: antiviral treatment options. Curr Opin Infect Dis. 2015;28(6):563–71. doi: 10.1097/QCO.0000000000000205 26524328 10.1097/QCO.0000000000000205

[60] Cicero TJ, Ellis MS. Abuse-Deterrent Formulations and the Prescription Opioid Abuse Epidemic in the United States: Lessons Learned From OxyContin. JAMA Psychiatry. 2015;72(5):424–30. doi: 10.1001/jamapsychiatry.2014.3043 25760692 10.1001/jamapsychiatry.2014.3043

[61] Dowell D, Zhang K, Noonan RK, Hockenberry JM. Mandatory Provider Review And Pain Clinic Laws Reduce The Amounts Of Opioids Prescribed And Overdose Death Rates. Health Aff (Millwood). 2016;35(10):1876–83. 27702962 10.1377/hlthaff.2016.0448 PMC6583870

[62] AASLD IDSA HCV Guidance Panel. Hepatitis C guidance: AASLD-IDSA recommendations for testing, managing, and treating adults infected with hepatitis C virus. Hepatology. 2015;62(3):932–54. doi: 10.1002/hep.27950 26111063 10.1002/hep.27950

[63] Martin NK, Vickerman P, Grebely J, Hellard M, Hutchinson SJ, Lima VD, et al Hepatitis C virus treatment for prevention among people who inject drugs: Modeling treatment scale-up in the age of direct-acting antivirals. Hepatology. 2013;58(5):1598–609. PubMed Central PMCID: PMCPMC3933734. doi: 10.1002/hep.26431 23553643 10.1002/hep.26431 PMC3933734

[64] Ogbuagu O, Bruce RD. Reaching the unreached: treatment as prevention as a workable strategy to mitigate HIV and its consequences in high-risk groups. Curr HIV/AIDS Rep. 2014;11(4):505–12. doi: 10.1007/s11904-014-0238-4 25342571 10.1007/s11904-014-0238-4

[65] Ahamad K, Hayashi K, Nguyen P, Dobrer S, Kerr T, Schutz CG, et al Effect of low-threshold methadone maintenance therapy for people who inject drugs on HIV incidence in Vancouver, BC, Canada: an observational cohort study. Lancet HIV. 2015;2(10):e445–50. PubMed Central PMCID: PMCPMC4675466. doi: 10.1016/S2352-3018(15)00129-0 26423652 10.1016/S2352-3018(15)00129-0 PMC4675466

[66] Magnani R, Sabin K, Saidel T, Heckathorn D. Review of sampling hard-to-reach and hidden populations for HIV surveillance. AIDS. 2005;19 Suppl 2:S67–72. 10.1097/01.aids.0000172879.20628.e1 15930843

[67] Apa ZL, Bai R, Mukherejee DV, Herzig CT, Koenigsmann C, Lowy FD, et al Challenges and strategies for research in prisons. Public Health Nurs. 2012;29(5):467–72. PubMed Central PMCID: PMCPMC3694772. doi: 10.1111/j.1525-1446.2012.01027.x 22924569 10.1111/j.1525-1446.2012.01027.x PMC3694772

[68] Ahrnsbrak R KL, and Harter R. 2015 National Survey on Drug Use and Health: Summary of the Effects of the 2015 NSDUH Questonnaire Redesign: Implications for Data Users [cited 2016 November 11]. Available from: http://www.samhsa.gov/data/sites/default/files/NSDUH-TrendBreak-2015.pdf. 30199192

[69] Saag MS. Editorial commentary: getting smart in how we pay for HCV drugs: KAOS vs CONTROL. Clin Infect Dis. 2015;61(2):169–70. doi: 10.1093/cid/civ221 25778748 10.1093/cid/civ221

[70] Cohen J. Pharmaceuticals. Advocates protest the cost of a hepatitis C cure. Science. 2013;342(6164):1302–3. doi: 10.1126/science.342.6164.1302 24337268 10.1126/science.342.6164.1302

[71] Davidson J. Obama anti-heroin strategy shifts focus to treatment from arrests: Washington Post; 2016 [updated March 29, 2016; cited 2016 July 29]. Available from: https://www.washingtonpost.com/news/powerpost/wp/2016/03/29/obama-unveils-anti-heroin-strategy/.

[72] Fact Sheet: Obama Administration Announces Additional Actions to Address the Prescription Opioid Abuse and Heroin Epidemic 2016 [cited 2016 July 29]. Available from: https://www.whitehouse.gov/the-press-office/2016/03/29/fact-sheet-obama-administration-announces-additional-actions-address.

