# Supplementary material for: The societal cost of heroin use disorder in the United States
Source: PLoS One. 2017 May 30;12(5):e0177323. doi: 10.1371/journal.pone.0177323 (PMC5448739; doi:10.1371/journal.pone.0177323)
Supplement: S1 Calculations — (DOCX) [file pone.0177323.s001.docx]

S 2 - Calculations for inputs derived from published sources:

Productivity Loss for Alive Heroin Users:

Proportion wages lost * median wages in 2014 (converted to 2015 dollars)

=17%[1] * $28,885.46[2] = $4,910.53

Overdose and Live Probability:

Number of heroin overdose patients who lived following overdose in 2009 divided by the total number of heroin users in 2009

=(83942+17989+8993+18774+3553+1934)[3]/399000[4]=0.339

Cost of HCV Treatment Per Patient:

Overall SVR (Sustained Virologic Response):

Successes/total = (120+386+271+799)/(125+396+281+835))=96.3%[5]

Cost per SVR in pooled real-world data was $84,700 (assumed to be in 2015 dollars).[5]

Cost per patient is $84,770 *0.963=$81,633.

Cost of Heroin Use Disorder Treatment

| Type of treatment | Census per type of treatment [1] | Proportion of total census treated for illicit drugs using treatment type[1] | Cost per person in 2007 dollars[1] | Weighted cost per treatment in 2007 dollars | Cost per person in 2015 dollars | Weighted cost per treatment in 2015 dollars |
| --- | --- | --- | --- | --- | --- | --- |
| Detoxification | 5,093 | 0.012 | $91,345.00 | $1,109.92 | $104,418.39 | $1,268.77 |
| Residential | 41,854 | 0.100 | $29,240.00 | $2,919.76 | $33,424.86 | $3,337.64 |
| Outpatient | 238,320 | 0.569 | $4,318.00 | $2,455.14 | $4,936.00 | $2,806.52 |
| Outpatient (Methadone) | 133,881 | 0.319 | $4,859.00 | $1,552.02 | $5,554.43 | $1,774.15 |
| Total Census | 419,148 | 1.000 | N/A | $8,036.84 | N/A | $9,187.08 |

Proportion of Incarcerated Users

200,000 incarcerated users[6] /(200,000 incarcerated users[6] + 808,000 non-incarcerated[7])=0.198

Proportion of Heroin users that gave birth to a baby

Proportion of pregnant female heroin users:

4 pregnant[8]/118 surveyed[8] = 3.390%

Proportion of females:

18942 female illicit drug users[8]/(24442 adult male illicit drug users[8] + 18942 adult female illicit drug users[8])= 43.6613%

Pregnant heroin users =43.6613%*3.390%=1.48%

1. US Department of Justice and National Drug Intelligence Center. The Economic Impact of Illicit Drug Use on American Society 2011 [cited 2016 January 29]. Available from: <http://www.justice.gov/archive/ndic/pubs44/44731/44731p.pdf>.

2. Social Security Administration. Measures Of Central Tendency For Wage Data - Average and Median Amounts of Net Compensation [cited 2016 July 18]. Available from: <https://www.ssa.gov/oact/cola/central.html>.

3. Inocencio TJ, Carroll NV, Read EJ, Holdford DA. The economic burden of opioid-related poisoning in the United States. Pain Med. 2013;14(10):1534-47. doi: 10.1111/pme.12183. PubMed PMID: 23841538.

4. Substance Abuse and Mental Health Services Administration. Results from the 2009 National Survey on Drug Use and Health: Volume I. Summary of National Findings 2009 [cited 2016 November 10]. Available from: <http://archive.samhsa.gov/data/NSDUH/2k9NSDUH/2k9Results.htm>.

5. Younossi ZMP, Haesuk; Gordon, Stuart C. ; Ferguson;John R.; Ahmed, Aijaz; Dieterich, Douglas; Saab, Sammy. Real-World Outcomes of Ledipasvir/Sofosbuvir in Treatment-Naïve Patients With Hepatitis C Am J Manag Care. 2016.

6. Boutwell AE, Nijhawan A, Zaller N, Rich JD. Arrested on heroin: a national opportunity. J Opioid Manag. 2007;3(6):328-32. PubMed PMID: 18290584.

7. Center for Behavioral Health Statistics and Quality. Key substance use and mental health indicators in the United States: Results from the 2015 National Survey on Drug Use and Health (HHS Publication No. SMA 16-4984, NSDUH Series H-51). 2016 [cited 2016 October 15]. Available from: <http://www.samhsa.gov/data/>.

8. Center for Behavioral Health Statistics and Quality Substance Abuse and Mental Health Services Administration. Results from the 2015 National Survey on Drug Use and Health: Detailed Tables [cited 2016 November 2]. Available from: <http://www.samhsa.gov/data/sites/default/files/NSDUH-DetTabs-2015/NSDUH-DetTabs-2015/NSDUH-DetTabs-2015.pdf>.
