# Supplementary material for: The societal cost of heroin use disorder in the United States
Source: PLoS One. 2017 May 30;12(5):e0177323. doi: 10.1371/journal.pone.0177323 (PMC5448739; doi:10.1371/journal.pone.0177323)
Supplement: S1 List — (DOCX) [file pone.0177323.s002.docx]

S1 – Model Assumptions:

- Population:
  - Population included in National Survey on Drug Use and Health (NSDUH) population (does not include prison/jail population) assumed not to overlap with people in prison due to heroin
  - For sensitivity analyses, 2010 chronic heroin user population (1.5 million) assumed to be equal to the same population in 2015.
- Use Disorder Treatment:
  - Not every user will be treated for use disorder
  - Cost of use disorder treatment assumed to be similar between incarcerated and general population
  - No distinction between use disorder treatment success and failure:
    - Productivity assumed to not return to that of a non-user at the end of treatment within the 1-year time line of the analytic model
- Infectious Diseases:
  - Acute infectious diseases not included as difficult to quantify frequency, duration, and/or treatment used.
  - Not everyone with an infectious disease will get treated for their disease(s)
    - Proportions of people treated assumed from those treated in commercial plans or those who developed active tuberculosis from latent tuberculosis
    - Costs used are average costs of active treatment (found from past studies so may not include the costs of the most updated treatments)
    - Healthcare costs of not treating patients for the infectious diseases were not part of the base model (assumed not to incur any healthcare costs because not getting treated)
  - HIV assumed to be main risk factor for tuberculosis. Thus, if the patient had HIV, no matter what other health conditions the patient had, their chance of having prevalent tuberculosis is the same as probability of having TB given HIV. If patients did not have HIV, no matter what other health conditions they had, then their chance of having prevalent tuberculosis is the same across all those disease states.
  - The probability of having HBV given other diseases was found in literature with as much detail as possible, then assumptions were made as necessary
    - The probability of having HBV given no other chronic infectious diseases (HIV, HCV) was assumed to have been higher than the probability of having HBV given that the user had at least 1 other infectious disease (HIV, HCV) due to the higher prevalence in all IDU than in those who were HCV or HIV positive
    - Otherwise assumed to be similar across HCV, HIV, and/or incarcerated patients
  - Cost of infectious disease treatment assumed to be similar between incarcerated and general population
  - No distinction between disease cure and failure because repercussions of either unlikely to be seen within 1-year time horizon
  - New infections or re-infections of a disease within this year are not captured in this prevalence-based model
    - Even if newly infected, unlikely to incur substantial healthcare costs within the first year of infection because the illness is unlikely to be diagnosed and/or treated
  - Treatment for a chronic illness are assumed to be independent of treatment for other illness(es), allowing for multiplication of treatment rates for individual chronic illnesses
- Overdose:
  - All users who overdose are treated for overdose
  - When a user overdoses, their healthcare costs for the overdose episode are the same whether they live or die
    - Users who live are weighted by the proportion of people who overdose and live
  - Overdose costs for user in prison assumed to be same as those out of prison
- Productivity:
  - Heroin users will have decreased productivity (built into increased costs to society) as compared to those who are not users
    - Per 2011 Economic Burden of Illicit Drug Use, illicit drug users will have 17-18% less productivity than their non-drug user counter parts depending on gender. It was assumed that they will cost 17% more (i.e. lose 17% of productivity) to society in our base model
  - If heroin users had not done heroin, they would have made the median wage per year of the US population in 2014 (converted to 2015 costs using CPI)
    - Mean wages were used as higher bound in sensitivity analysis
  - Productivity losses will be the same across terminal nodes as assumed not to be substantially affected by the infectious diseases
  - When users die or become incarcerated (whether they live or die in prison), they lose all of the median yearly wage for a year
- Crime
  - The heroin user is assumed to be similar to an average illicit drug user, so allows for the use of average crime cost incurred by an illicit drug user to be translated to the heroin user
- Incarceration:
  - Average cost of incarceration used and only for those incarcerated
  - Crime costs assumed to be $0 while users are in prison
- Mortality:
  - Drug related deaths far outweigh deaths due to infectious disease, thus, all patients that died were assumed to have died of an overdose
  - Mortality is different for patients on opioid use disorder treatment vs. not on treatment
- Cost of heroin to user:
  - Incarcerated users were assumed not to purchase heroin using currency
- Neonatal Abstinence Syndrome (NAS)
  - All women pregnant at time of NSDUH survey gave birth to a single child that required treatment for NAS in 2015
  - All babies born to mothers who were using heroin were treated for NAS
