# Supplementary material for: The societal cost of heroin use disorder in the United States
Source: PLoS One. 2017 May 30;12(5):e0177323. doi: 10.1371/journal.pone.0177323 (PMC5448739; doi:10.1371/journal.pone.0177323)
Supplement: S1 Table — (DOCX) [file pone.0177323.s003.docx]

S3 – Conversion of Costs Found in Literature to 2015 Costs using Consumer Price Index

| Variable | Non-incarcerated heroin users | | | Incarcerated heroin users | | |
| --- | --- | --- | --- | --- | --- | --- |
|  | Original citation cost [ref] | Original citation year | Updated to 2015 cost | Original citation cost [ref] | Original citation year | Updated to 2015 cost |
| Cost of opioid use disorder treatment (calculated) | $8,036.84[1] | 2007 | $9,187.08 | $8,036.84[1] | 2007 | $9,187.08 |
| Cost of HIV treatment | $19,912[2] | 2006 | $23,681.71 | $19,912[2] | 2006 | $23,681.71 |
| Cost of HCV treatment | $81,633.51[3] | 2015 | $81,633.51 | $81,633.51[3] | 2015 | $81,633.51 |
| Cost of HBV treatment | $20,206[4] | 1999 | $28,817.62 | $20,206[4] | 1999 | $28,817.62 |
| Cost of TB treatment | $495.21[5] | 2008 | $545.15 | $495.21[5] | 2008 | $545.15 |
| Cost to treat each overdose | $3,198[6] | 2011 | $3369.71 | $3,198[6] | 2011 | $3369.71 |
| Cost of crime committed by each user | $5944.93[1] | 2007 | $6795.77 | N/A | N/A | N/A |
| Cost of NAS treatment | $66,700[7] | 2012 | $68,856.48 | $66,700[7] | 2012 | $68,856.48 |
| Cost of Heroin to user | $1457/month  =$17,484/year[8] | 2010 | $19,004.32 | N/A | N/A | N/A |
| Cost of incarceration | N/A | N/A | N/A | $30,619.85[9] | 2014 | $30,656.20 |
| Lost productivity by non-incarcerated user who lives | $4,904.70[1, 10] | 2014 | $4,910.53 | N/A | N/A | N/A |
| Lost productivity by non-incarcerated user who dies | $28,851.20[1, 10] | 2014 | $28,885.46 | N/A | N/A | N/A |

1. US Department of Justice and National Drug Intelligence Center. The Economic Impact of Illicit Drug Use on American Society 2011 [cited 2016 January 29]. Available from: <http://www.justice.gov/archive/ndic/pubs44/44731/44731p.pdf>.

2. Gebo KA, Fleishman JA, Conviser R, Hellinger J, Hellinger FJ, Josephs JS, et al. Contemporary costs of HIV healthcare in the HAART era. AIDS. 2010;24(17):2705-15. doi: 10.1097/QAD.0b013e32833f3c14. PubMed PMID: 20859193; PubMed Central PMCID: PMCPMC3551268.

3. Younossi ZMP, Haesuk; Gordon, Stuart C. ; Ferguson;John R.; Ahmed, Aijaz; Dieterich, Douglas; Saab, Sammy. Real-World Outcomes of Ledipasvir/Sofosbuvir in Treatment-Naïve Patients With Hepatitis C Am J Manag Care. 2016.

4. Lavanchy D. Hepatitis B virus epidemiology, disease burden, treatment, and current and emerging prevention and control measures. J Viral Hepat. 2004;11(2):97-107. PubMed PMID: 14996343.

5. Holland DP, Sanders GD, Hamilton CD, Stout JE. Costs and cost-effectiveness of four treatment regimens for latent tuberculosis infection. Am J Respir Crit Care Med. 2009;179(11):1055-60. doi: 10.1164/rccm.200901-0153OC. PubMed PMID: 19299495; PubMed Central PMCID: PMCPMC2689913.

6. Inocencio TJ, Carroll NV, Read EJ, Holdford DA. The economic burden of opioid-related poisoning in the United States. Pain Med. 2013;14(10):1534-47. doi: 10.1111/pme.12183. PubMed PMID: 23841538.

7. Patrick SW, Davis MM, Lehmann CU, Cooper WO. Increasing incidence and geographic distribution of neonatal abstinence syndrome: United States 2009 to 2012. J Perinatol. 2015;35(8):650-5. doi: 10.1038/jp.2015.36. PubMed PMID: 25927272; PubMed Central PMCID: PMCPMC4520760.

8. Kilmer B ES, Caulkins JP, et al. What America's Users Spend on Illicit Drugs: 2000 through 2010 2014 [cited 2016 July 29]. Available from: <http://www.rand.org/pubs/research_reports/RR534.html>.

9. Samuels J, CE. Annual Determination of Average Cost of Incarceration 2015 [cited 2016 July 27]. Available from: <https://www.federalregister.gov/articles/2015/03/09/2015-05437/annual-determination-of-average-cost-of-incarceration>.

10. Social Security Administration. Measures Of Central Tendency For Wage Data - Average and Median Amounts of Net Compensation [cited 2016 July 18]. Available from: <https://www.ssa.gov/oact/cola/central.html>.
