# Supplementary material for: The societal cost of heroin use disorder in the United States
Source: PLoS One. 2017 May 30;12(5):e0177323. doi: 10.1371/journal.pone.0177323 (PMC5448739; doi:10.1371/journal.pone.0177323)
Supplement: S2 Table — (DOCX) [file pone.0177323.s004.docx]

S 4: Results - Costs by Type; Shown by Heroin User Classification (Population Total and by Incarceration Status)

|  | Average Cost Per Type of User | | | | | | Total Cost |
| --- | --- | --- | --- | --- | --- | --- | --- |
|  | All heroin users | | Non-incarcerated heroin users | | Incarcerated heroin users | | Entire heroin-using population |
| Type of cost | Cost per user | Percentage of overall cost | Cost per user | Percentage of overall cost | Cost per user | Percentage of overall cost | Total Costs  (in millions) |
| Overall cost per user | $50,799 | 100.0% | $44,950 | 100.0% | $74,428 | 100.0% | $51,206 |
| Productivity Costs | $9,809 | 19.3% | $5,087 | 11.3% | $28,885 | 38.8% | $9,888 |
| Non-Productivity Costs | $40,990 | 80.7% | $39,863 | 88.7% | $45,542 | 61.2% | $41,318 |
| Heroin Use Disorder Treatment Costs | $1,067 | 2.1% | $1,011 | 2.2% | $1,295 | 1.7% | $1,076 |
| HIV Treatment Costs | $890 | 1.8% | $506 | 1.1% | $2,445 | 3.3% | $897 |
| HCV Treatment Costs | $9,811 | 19.3% | $10,073 | 22.4% | $8,755 | 11.8% | $9,890 |
| HBV Treatment Costs | $270 | 0.5% | $286 | 0.6% | $205 | 0.3% | $272 |
| TB Treatment Costs | $10 | 0.0% | $10 | 0.0% | $9 | 0.0% | $10 |
| Overdose Treatment Costs | $1,158 | 2.3% | $1,159 | 2.6% | $1,158 | 1.6% | $1,168 |
| Crime Costs | $5,447 | 10.7% | $6,796 | 15.1% | $0 | 0.0% | $5,491 |
| Incarceration Costs | $6,083 | 12.0% | $0 | 0.0% | $30,656 | 41.2% | $6,131 |
| NAS Treatment Costs | $1,019 | 2.0% | $1,019 | 2.3% | $1,019 | 1.4% | $1,027 |
| Cost of Heroin to Users | $15,234 | 30.0% | $19,004 | 42.3% | $0 | 0.0% | $15,355 |
