# Supplementary material for: The societal cost of heroin use disorder in the United States
Source: PLoS One. 2017 May 30;12(5):e0177323. doi: 10.1371/journal.pone.0177323 (PMC5448739; doi:10.1371/journal.pone.0177323)
Supplement: S3 Table — (DOCX) [file pone.0177323.s005.docx]

S 5 – Full One-Way Sensitivity Analyses Results

| **Variable Name** | **Base Value** | **Low Input** | **High Input** | **Lower Bound (cost/user)** | **Lower bound total heroin cost (in millions)** | **Lower Bound % of Base Case** | **Higher Bound (cost/user)** | **Higher bound total heroin cost (in millions)** | **Upper Bound % of Base Case** |
| --- | --- | --- | --- | --- | --- | --- | --- | --- | --- |
| Number of heroin users | 1,008,000[1, 2] | 324,000[2] | 1,500,000[3] | $50,799.14 | $16,458.92 | 32.14% | $50,799.14 | $76,198.71 | 148.81% |
| Cost of HCV Treatment | $81,633.51[4] | $18,976.95[5] | $101,380.00[4] | $43,268.70 | $43,614.85 | 85.18% | $53,172.39 | $53,597.77 | 104.67% |
| Cost of incarceration | $23,681.71[6] | $15,872.80[7] | $65,299.89[7] | $47,865.92 | $48,248.85 | 94.23% | $57,672.89 | $58,134.27 | 113.53% |
| Proportion of users in Prison | 0.1984[1, 2] | 0.1984[1, 2] | 0.36[1] | $50,798.76 | $51,205.15 | 100.00% | $55,562.32 | $56,006.82 | 109.38% |
| Lost productivity by incarcerated user | $28,885.46[8] | $28,885.46[8] | $44,622.10[8] | $50,799.14 | $51,205.53 | 100.00% | $53,899.02 | $54,330.21 | 106.10% |
| Lost productivity by non-incarcerated users outside of prison | $4,910.53[8, 9] | $4,910.53[8, 9] | $7,585.76[8, 9] | $50,799.14 | $51,205.53 | 100.00% | $52,927.76 | $53,351.18 | 104.19% |
| Cost of HIV Treatment | $23,681.71[10] | $18,520.02[11] | $54,903.49[11] | $50,605.08 | $51,009.92 | 99.62% | $51,972.95 | $52,388.74 | 102.31% |
| Ratio of incarcerated to non-incarcerated users treated for health condition | 1.00 | 0.75^^^ | 1.25^^^ | $50,168.83 | $50,570.18 | 98.76% | $51,429.44 | $51,840.88 | 101.24% |
| Proportion of non-incarcerated users with HIV | 0.07[12] | 0.07[12] | 0.224[13] | $50,799.14 | $51,205.53 | 100.00% | $51,744.27 | $52,158.23 | 101.86% |
| Proportion of non-incarcerated users treated for heroin use disorder | 0.110[14] | 0.110[14] | 0.197[15] | $50,799.14 | $51,205.53 | 100.00% | $51,429.41 | $51,840.85 | 101.24% |
| Cost of Neonatal Abstinence Syndrome treatment | $68,856.48[16] | $54,230.37[17] | $96,419.71[16] | $50,582.67 | $50,987.33 | 99.57% | $51,207.07 | $51,616.73 | 100.80% |
| Cost of heroin use disorder treatment | $9,187.08[9] | $3,986.06[18] | $9,187.08[9] | $50,195.04 | $50,596.60 | 98.81% | $50,799.14 | $51,205.53 | 100.00% |
| Proportion of non-incarcerated users treated for HIV | 0.305[19] | 0.305[19] | 0.408[20] | $50,799.14 | $51,205.53 | 100.00% | $50,936.00 | $51,343.49 | 100.27% |
| Lost productivity by dead user | $28,885.46[8] | $28,885.46[8] | $44,622.10[8] | $50,799.14 | $51,205.53 | 100.00% | $50,914.61 | $51,321.93 | 100.23% |
| Proportion of incarcerated users treated for HIV | 0.333[21] | 0.333[21] | 0.408[20] | $50,799.14 | $51,205.53 | 100.00% | $50,908.38 | $51,315.65 | 100.22% |
| Proportion of incarcerated users treated for use disorder | 0.141[22] | 0.141[22] | 0.17[23] | $50,799.14 | $51,205.53 | 100.00% | $50,851.93 | $51,258.74 | 100.10% |
| Proportion of non-incarcerated users treated for HBV | 0.14[24] | 0.14[24] | 0.17[24] | $50,799.14 | $51,205.53 | 100.00% | $50,848.32 | $51,255.11 | 100.10% |
| Risk of dying while non-incarcerated and on use disorder treatment | 0.0023[25] | 0.0023[25] | 0.016[26, 27] | $50,799.14 | $51,205.53 | 100.00% | $50,830.79 | $51,237.44 | 100.06% |
| Cost of treatment for Tuberculosis | $545.15[28] | $545.15[28] | $1,681.37[28] | $50,799.14 | $51,205.53 | 100.00% | $50,819.76 | $51,226.32 | 100.04% |
| Proportion of incarcerated users receiving HBV treatment | 0.14[24] | 0.14[24] | 0.17[24] | $50,799.14 | $51,205.53 | 100.00% | $50,807.84 | $51,214.30 | 100.02% |
| Risk of dying while incarcerated and on use disorder treatment | 0.0023[25] | 0.0023[25] | 0.016[26, 27] | $50,799.14 | $51,205.53 | 100.00% | $50,799.99 | $51,206.39 | 100.00% |
| Probability of having TB given patient is HIV-positive while incarcerated | 0.16[29] | 0.16[29] | 0.3[30] | $50,799.14 | $51,205.53 | 100.00% | $50,799.37 | $51,205.76 | 100.00% |
| Probability of having TB given patient is HIV-positive while non-incarcerated | 0.16[29] | 0.16[29] | 0.3[30] | $50,799.14 | $51,205.53 | 100.00% | $50,799.36 | $51,205.75 | 100.00% |

^^^Assumption

References

1. Boutwell AE, Nijhawan A, Zaller N, Rich JD. Arrested on heroin: a national opportunity. J Opioid Manag. 2007;3(6):328-32. PubMed PMID: 18290584.

2. Center for Behavioral Health Statistics and Quality. Key substance use and mental health indicators in the United States: Results from the 2015 National Survey on Drug Use and Health (HHS Publication No. SMA 16-4984, NSDUH Series H-51). 2016 [cited 2016 October 15]. Available from: <http://www.samhsa.gov/data/>.

3. Kilmer B ES, Caulkins JP, et al. What America's Users Spend on Illicit Drugs: 2000 through 2010 2014 [cited 2016 July 29]. Available from: <http://www.rand.org/pubs/research_reports/RR534.html>.

4. Younossi ZMP, Haesuk; Gordon, Stuart C. ; Ferguson;John R.; Ahmed, Aijaz; Dieterich, Douglas; Saab, Sammy. Real-World Outcomes of Ledipasvir/Sofosbuvir in Treatment-Naïve Patients With Hepatitis C Am J Manag Care. 2016.

5. Solomon M, Bonafede M, Pan K, Wilson K, Beam C, Chakravarti P, et al. Direct medical care costs among pegylated interferon plus ribavirin-treated and untreated chronic hepatitis C patients. Dig Dis Sci. 2011;56(10):3024-31. doi: 10.1007/s10620-011-1802-z. PubMed PMID: 21717127.

6. Samuels J, CE. Annual Determination of Average Cost of Incarceration 2015 [cited 2016 July 27]. Available from: <https://www.federalregister.gov/articles/2015/03/09/2015-05437/annual-determination-of-average-cost-of-incarceration>.

7. Henrichson CD, R. The Price of Prisons: What Incarceration Costs Taxpayers. New York: Vera Institute of Justice; 2012 [updated July 20, 2012; cited 2016 October 16]. Available from: <http://archive.vera.org/sites/default/files/resources/downloads/price-of-prisons-updated-version-021914.pdf>.

8. Social Security Administration. Measures Of Central Tendency For Wage Data - Average and Median Amounts of Net Compensation [cited 2016 July 18]. Available from: <https://www.ssa.gov/oact/cola/central.html>.

9. US Department of Justice and National Drug Intelligence Center. The Economic Impact of Illicit Drug Use on American Society 2011 [cited 2016 January 29]. Available from: <http://www.justice.gov/archive/ndic/pubs44/44731/44731p.pdf>.

10. Gebo KA, Fleishman JA, Conviser R, Hellinger J, Hellinger FJ, Josephs JS, et al. Contemporary costs of HIV healthcare in the HAART era. AIDS. 2010;24(17):2705-15. doi: 10.1097/QAD.0b013e32833f3c14. PubMed PMID: 20859193; PubMed Central PMCID: PMCPMC3551268.

11. Schackman BR, Fleishman JA, Su AE, Berkowitz BK, Moore RD, Walensky RP, et al. The lifetime medical cost savings from preventing HIV in the United States. Med Care. 2015;53(4):293-301. doi: 10.1097/MLR.0000000000000308. PubMed PMID: 25710311; PubMed Central PMCID: PMCPMC4359630.

12. Spiller MW, Broz D, Wejnert C, Nerlander L, Paz-Bailey G, Centers for Disease C, et al. HIV infection and HIV-associated behaviors among persons who inject drugs--20 cities, United States, 2012. MMWR Morb Mortal Wkly Rep. 2015;64(10):270-5. PubMed PMID: 25789742.

13. Mathers BM, Degenhardt L, Phillips B, Wiessing L, Hickman M, Strathdee SA, et al. Global epidemiology of injecting drug use and HIV among people who inject drugs: a systematic review. Lancet. 2008;372(9651):1733-45. doi: 10.1016/S0140-6736(08)61311-2. PubMed PMID: 18817968.

14. National Institute on Drug Abuse. DrugFacts: Nationwide Trends [updated June 2015; cited 2016 Feburary 1]. Available from: <http://www.drugabuse.gov/publications/drugfacts/nationwide-trends>.

15. Saloner B, Karthikeyan S. Changes in Substance Abuse Treatment Use Among Individuals With Opioid Use Disorders in the United States, 2004-2013. JAMA. 2015;314(14):1515-7. doi: 10.1001/jama.2015.10345. PubMed PMID: 26462001.

16. Patrick SW, Davis MM, Lehmann CU, Cooper WO. Increasing incidence and geographic distribution of neonatal abstinence syndrome: United States 2009 to 2012. J Perinatol. 2015;35(8):650-5. doi: 10.1038/jp.2015.36. PubMed PMID: 25927272; PubMed Central PMCID: PMCPMC4520760.

17. Patrick SW, Schumacher RE, Benneyworth BD, Krans EE, McAllister JM, Davis MM. Neonatal abstinence syndrome and associated health care expenditures: United States, 2000-2009. JAMA. 2012;307(18):1934-40. doi: 10.1001/jama.2012.3951. PubMed PMID: 22546608.

18. Baser O, Chalk M, Fiellin DA, Gastfriend DR. Cost and utilization outcomes of opioid-dependence treatments. Am J Manag Care. 2011;17 Suppl 8:S235-48. PubMed PMID: 21761950.

19. Westergaard RP, Hess T, Astemborski J, Mehta SH, Kirk GD. Longitudinal changes in engagement in care and viral suppression for HIV-infected injection drug users. AIDS. 2013;27(16):2559-66. doi: 10.1097/QAD.0b013e328363bff2. PubMed PMID: 23770493; PubMed Central PMCID: PMCPMC3795966.

20. Sherman KE, Rockstroh J, Thomas D. Human immunodeficiency virus and liver disease: An update. Hepatology. 2015;62(6):1871-82. doi: 10.1002/hep.28150. PubMed PMID: 26340591; PubMed Central PMCID: PMCPMC4681629.

21. Wakeman SE, Rich JD. HIV treatment in US prisons. HIV Ther. 2010;4(4):505-10. PubMed PMID: 20953349; PubMed Central PMCID: PMCPMC2953806.

22. Mumola CJK, J.C. Drug Use and Dependence, State and Federal Prisoners, 2004 2006 [cited 2016 July 29]. Available from: <http://www.bjs.gov/content/pub/pdf/dudsfp04.pdf>.

23. National Institute on Drug Abuse. Drug Addiction Treatment in the Criminal Justice System [cited 2016 May 10]. Available from: ht t ps : / / www. dr ug abus e. g ov/ r el at ed­ t opi c s / c r i mi nal ­ j us t i c e/ dr ug ­ addi c t i on­ t r eat ment ­ i n­ c r i mi nal ­ j us t i c e­ s ys t em.

24. Sarkar M, Shvachko VA, Ready JB, Pauly MP, Terrault NA, Peters MG, et al. Characteristics and management of patients with chronic hepatitis B in an integrated care setting. Dig Dis Sci. 2014;59(9):2100-8. doi: 10.1007/s10620-014-3142-2. PubMed PMID: 24728968; PubMed Central PMCID: PMCPMC4149592.

25. Evans E, Li L, Min J, Huang D, Urada D, Liu L, et al. Mortality among individuals accessing pharmacological treatment for opioid dependence in California, 2006-10. Addiction. 2015;110(6):996-1005. doi: 10.1111/add.12863. PubMed PMID: 25644938; PubMed Central PMCID: PMCPMC4452110.

26. Lipari RNaH, Arthur Trends in Heroin Use in the United States: 2002 TO 2013. 2015.

27. H. H, LH. C, M. W. Drug-poisoning Deaths Involving Heroin: United States, 2000–2013 2015. Available from: https://www.cdc.gov/nchs/data/databriefs/db190.pdf.

28. Holland DP, Sanders GD, Hamilton CD, Stout JE. Costs and cost-effectiveness of four treatment regimens for latent tuberculosis infection. Am J Respir Crit Care Med. 2009;179(11):1055-60. doi: 10.1164/rccm.200901-0153OC. PubMed PMID: 19299495; PubMed Central PMCID: PMCPMC2689913.

29. Golub JE, Astemborski J, Ahmed M, Cronin W, Mehta SH, Kirk GD, et al. Long-term effectiveness of diagnosing and treating latent tuberculosis infection in a cohort of HIV-infected and at-risk injection drug users. J Acquir Immune Defic Syndr. 2008;49(5):532-7. doi: 10.1097/QAI.0b013e31818d5c1c. PubMed PMID: 18989223; PubMed Central PMCID: PMCPMC2637943.

30. Getahun H, Gunneberg C, Granich R, Nunn P. HIV infection-associated tuberculosis: the epidemiology and the response. Clin Infect Dis. 2010;50 Suppl 3:S201-7. doi: 10.1086/651492. PubMed PMID: 20397949.
